# Caveolin-1 mediated uptake via langerin restricts HIV-1 infection in human Langerhans cells

**DOI:** 10.1186/s12977-014-0123-7

**Published:** 2014-12-31

**Authors:** Linda M van den Berg, Carla M S Ribeiro, Esther M Zijlstra-Willems, Lot de Witte, Donna Fluitsma, Wikky Tigchelaar, Vincent Everts, Teunis B H Geijtenbeek

**Affiliations:** Department of Experimental Immunology, Academic Medical Center, University of Amsterdam, Meibergdreef 9, 1105 AZ Amsterdam, The Netherlands; Department of Psychiatry, University Medical Center Utrecht, Utrecht, The Netherlands; Department of Molecular Cell Biology and Immunology, VU University Medical Center, Amsterdam, The Netherlands; Department of Cell Biology & Histology, Academic Medical Center, University of Amsterdam, Amsterdam, The Netherlands; Department of Oral Cell Biology, Academic Center for Dentistry Amsterdam, University of Amsterdam and VU University Amsterdam, Amsterdam, The Netherlands

**Keywords:** HIV-1 restriction, Caveolin-1, Langerhans cells, Langerin, Birbeck granules, Caveolar uptake, Clathrin

## Abstract

**Background:**

Human Langerhans cells (LCs) reside in foreskin and vaginal mucosa and are the first immune cells to interact with HIV-1 during sexual transmission. LCs capture HIV-1 through the C-type lectin receptor langerin, which routes the virus into Birbeck granules (BGs), thereby preventing HIV-1 infection. BGs are langerin-positive organelles exclusively present in LCs, however, their origin and function are unknown.

**Results:**

Here, we not only show that langerin and caveolin-1 co-localize at the cell membrane and in vesicles but also that BGs are langerin/caveolin-1-positive vesicles are linked to the lysosomal degradation pathway in LCs. Moreover, inhibition of caveolar endocytosis in primary LCs abrogated HIV-1 sequestering into langerin^+^ caveolar structures. Notably, both inhibition of caveolar uptake and silencing of caveolar structure protein caveolin-1 resulted in increased HIV-1 integration and subsequent infection. In contrast, inhibition of clathrin-mediated endocytosis did not affect HIV-1 integration, even though HIV-1 uptake was decreased, suggesting that clathrin-mediated endocytosis is not involved in HIV-1 restriction in LCs.

**Conclusions:**

Thus, our data strongly indicate that BGs belong to the caveolar endocytosis pathway and that caveolin-1 mediated HIV-1 uptake is an intrinsic restriction mechanism present in human LCs that prevents HIV-1 infection. Harnessing this particular internalization pathway has the potential to facilitate strategies to combat HIV-1 transmission.

**Electronic supplementary material:**

The online version of this article (doi:10.1186/s12977-014-0123-7) contains supplementary material, which is available to authorized users.

## Background

Langerhans cells (LCs) are a specialized subset of antigen presenting cells in the epidermis of the skin and mucosal tissues of the vagina and foreskin. They provide a barrier against entry of pathogens, thereby protecting against disease [1-3]. Due to their location, LCs are among the first immune cells that encounter HIV-1 in genital tissue during sexual transmission [4,5]. LCs are not efficiently infected with HIV-1 and do not transmit virus to T cells [3]. However, Toll-like receptor activation and high viral loads enhance HIV-1 transmission by human LCs [6-8]. LCs express the C-type lectin receptor (CLR) langerin that captures HIV-1, which is subsequently internalized into Birbeck granules (BGs), where the virus is thought to be degraded [3]. Little is known about the function of BGs and how it contributes to limiting HIV-1 infection. Although conflicting theories exist regarding the origin and function of BGs [9], it is clear that the expression of functional langerin is a prerequisite for the formation of BGs [10,11]. Ectopic expression of langerin in cell lines induces BG formation and antibodies against langerin are internalized into BGs [12,13]. Langerin-mediated internalization is thought to occur through classical clathrin-coated endosomal uptake [14]. However, the cytoplasmic domain of langerin does not contain ‘classic’ internalization motifs that are associated with clathrin binding or formation of the coated pits, such as a double-tyrosine or tri-leucine motif [10,15,16]. In addition, BGs have been proposed to be subdomains of the endosomal recycling compartment and recent studies show that caveolin-1 not only overlaps with endocytic recycling compartments in epithelial cells but also contributes to LCs ability to cross-present antigens to CD8^+^ T cells. [14,17,18]. HIV-1 internalization into BGs is important to the anti-viral function of LCs. We investigated the internalization route of HIV-1 and the role of caveolin-1 dependent internalization in protection against HIV-1 infection in LCs. Here, we show that BGs are caveolin-1-positive vesicles and that caveolin-1 prevents HIV-1 infection in human Langerhans cells.

## Results and discussion

### Langerin co-localizes with caveolin-1

**Figure 1:**
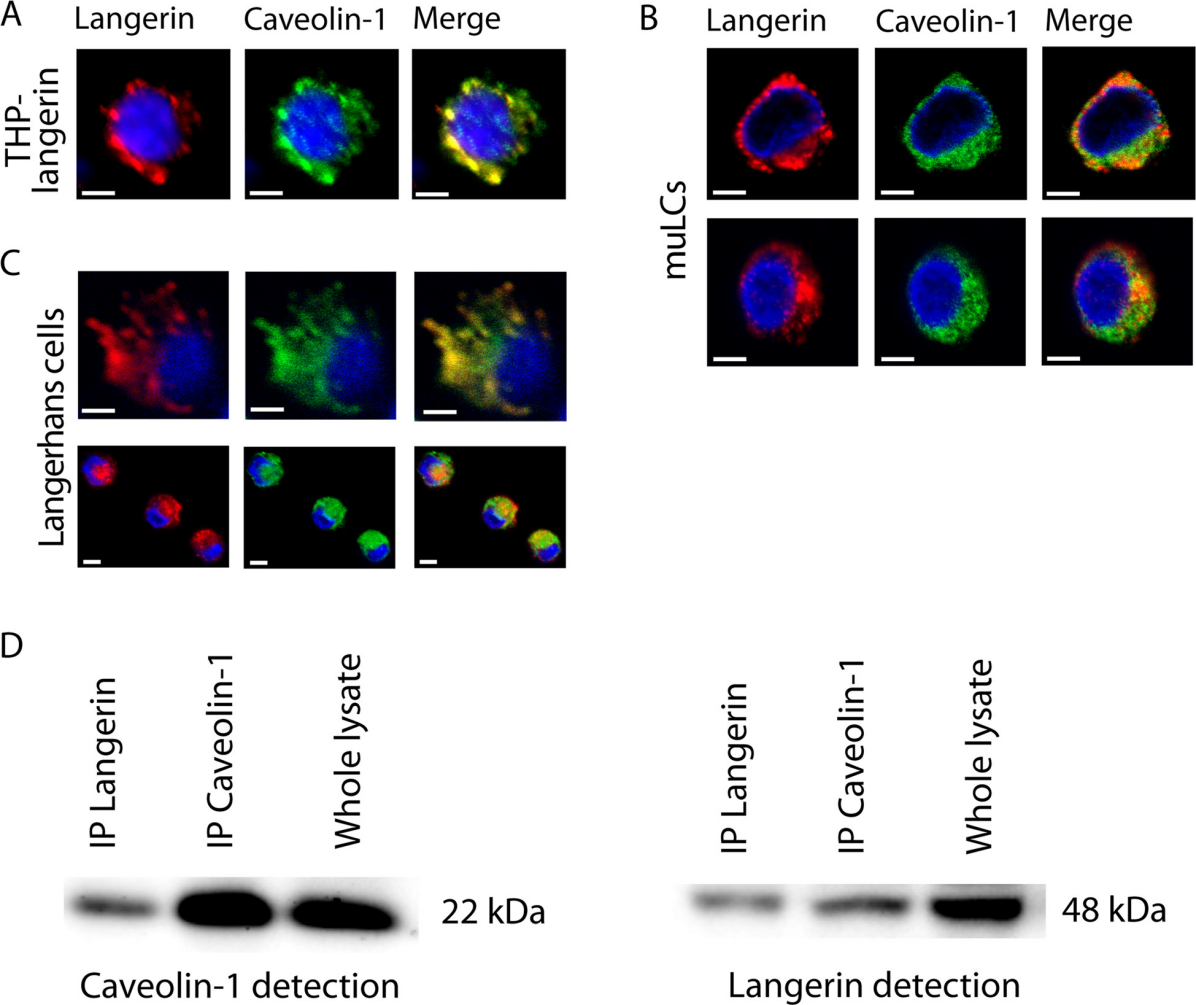
**Langerin co-localizes with caveolin-1 in steady state.** Confocal scanning laser microscopic analyses for THP-langerin cell line **(A)**, MUTZ3-LC cell line (MUTZ-LCs, **B)** and human LCs **(C)** for langerin and caveolin-1 in steady-state condition in permeablized cells; bars represent 10 μm **(A, B, C)**. Langerin and caveolin-1 were immuno-precipitated from LC cell lysates and immuno-blotted with antibodies against caveolin-1 or langerin **(D)**. These data are representative of at least 3 donors or 3 independent experiments.

Lipid raft internalization is the major internalization route besides clathrin-mediated endocytosis. Caveolar internalization occurs via lipid rafts and is dependent on the integral membrane molecule caveolin-1 [19,20]. Caveolae are small cholesterol-rich invaginations in the plasma membrane that can form caveolar vesicles [21,22], which fuse with late endosomes and lysosomes [23]. We investigated whether langerin co-localized with the major caveolar structural protein caveolin-1 in primary human LCs, MUTZ3-derived LC cells (MUTZ-LCs) and a langerin-transduced cell line (THP-langerin). Under steady-state conditions, caveolin-1 and langerin partially co-localized in THP-langerin, MUTZ-LCs as well as in primary LCs as shown by confocal immunofluorescence microscopy (Figure 1A,B,C). To further investigate co-localization in lipid rafts we performed co-immunoprecipitation assays from lysates of primary LCs. Caveolin-1 co-immunoprecipitated with langerin and vice versa (Figure 1D), supporting our imaging data that langerin and caveolin-1 co-localize in LCs.

### HIV-1 uptake by LCs depends on caveolin-1

**Figure 2:**
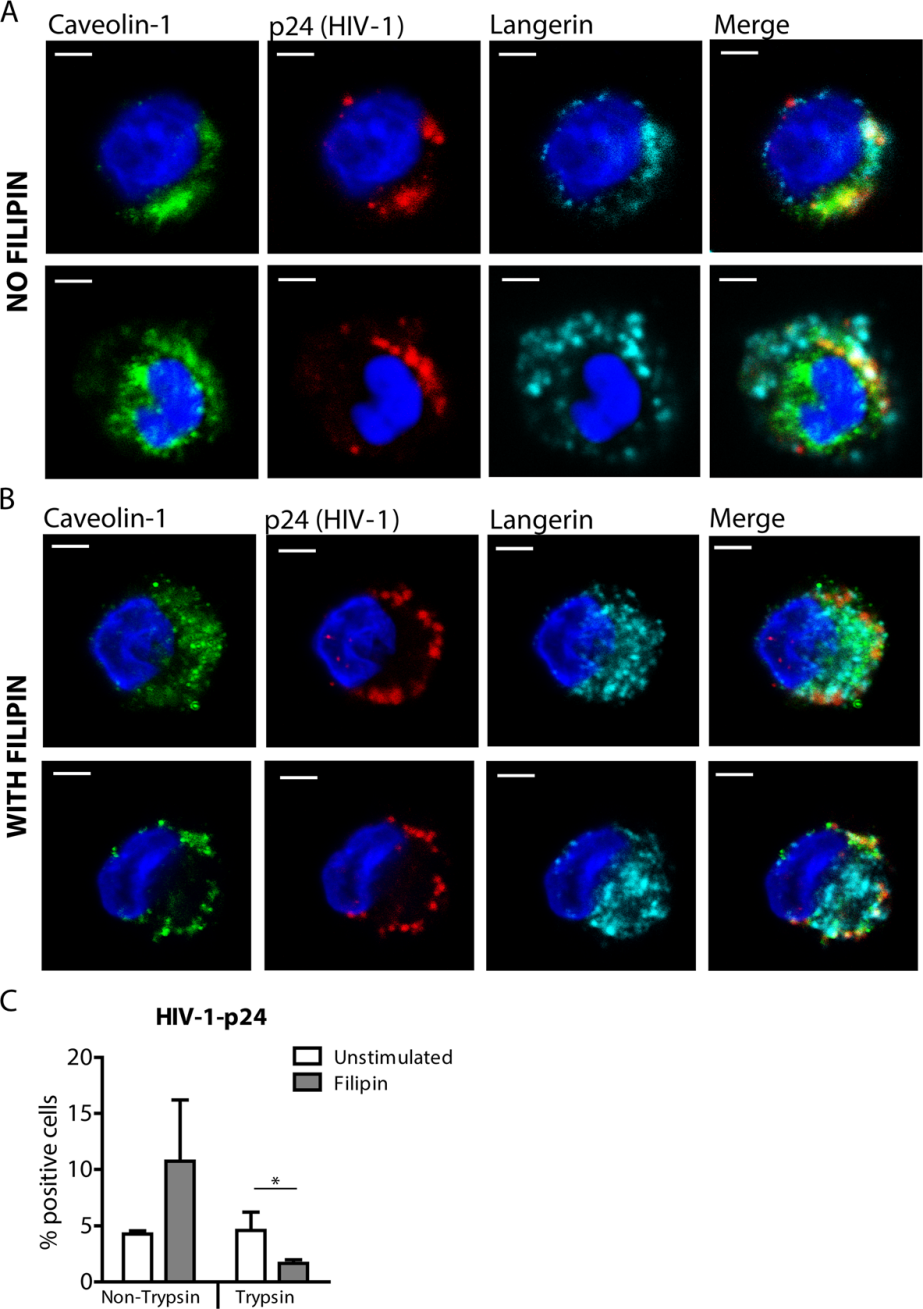
**Langerin, caveolin-1 and HIV-1 partly co-localize in the same organelle.** LCs were incubated with HIV-1 for 4 h in the absence **(A)** or presence of the caveolar inhibitor filipin (1 μg/ml, B) LCs were stained for p24-HIV-1, langerin and caveolin-1; representative for 3 donors; bars represent 10 μm **(A, B)**. LCs were incubated with HIV-1 for 4 h in the absence or presence of filipin (1 μg/ml). Cells were treated with Trypsin-EDTA (0.05%) or left untreated as control. HIV-1 uptake was quantified by permeabilizing cells and measuring subsequent HIV-1-p24 content by flow cytometry. n = 3 paired students *t*-test; *p < 0.05; SD and mean are depicted **(C)**.

Langerin expressed by LCs captures HIV-1 for internalization into BGs [3]. We therefore investigated whether caveolin-1-mediated internalization is involved in HIV-1 uptake using filipin, an inhibitor of caveolar uptake [24,25]. Filipin impairs caveolae invaginations and caveolar endocytosis [26-28]. Primary LCs were incubated with HIV-1 for 4 hours and internalization was assessed by confocal immunofluorescence microscopy. Our data show that internalized HIV-1 partly co-localized with langerin as well as caveolin-1 (Figure 2A). Notably, our data suggest that HIV-1 uptake is mediated by caveolar endocytosis, since we observed increased HIV-1 staining at the cell-surface and less intracellular HIV-1 in the presence of filipin (Figure 2B). Filipin treatment increased surface expression of langerin (Additional file 1a, b), strongly supporting a role for caveolin-mediated uptake in langerin trafficking. Next, we quantified HIV-1 uptake by flow cytometry. LCs were incubated with HIV-1 for 4 hours and cell-surface bound HIV-1 was removed by trypsin. LCs efficiently internalized HIV-1 since trypsin treatment did not affect HIV-1 staining (Figure 2C). Filipin treatment increased HIV-1 binding to LCs compared to untreated LCs (Figure 2C), which might be due to increased expression of langerin on the cell-surface due to inhibition of caveolin-mediated endocytosis (Additional file 1a, b). Notably, filipin treatment significantly decreased HIV-1 internalization by LCs, since removal of surface-bound virions from filipin-treated LCs by trypsin significantly decreased HIV-1 staining compared to untreated LCs. These data strongly support a role for caveolin-mediated endocytosis of HIV-1 by LCs. These data strongly suggest that caveolin-1 is necessary for langerin-mediated HIV-1 internalization by LCs.

### Birbeck granules belong to the caveolar endocytosis pathway

**Figure 3:**
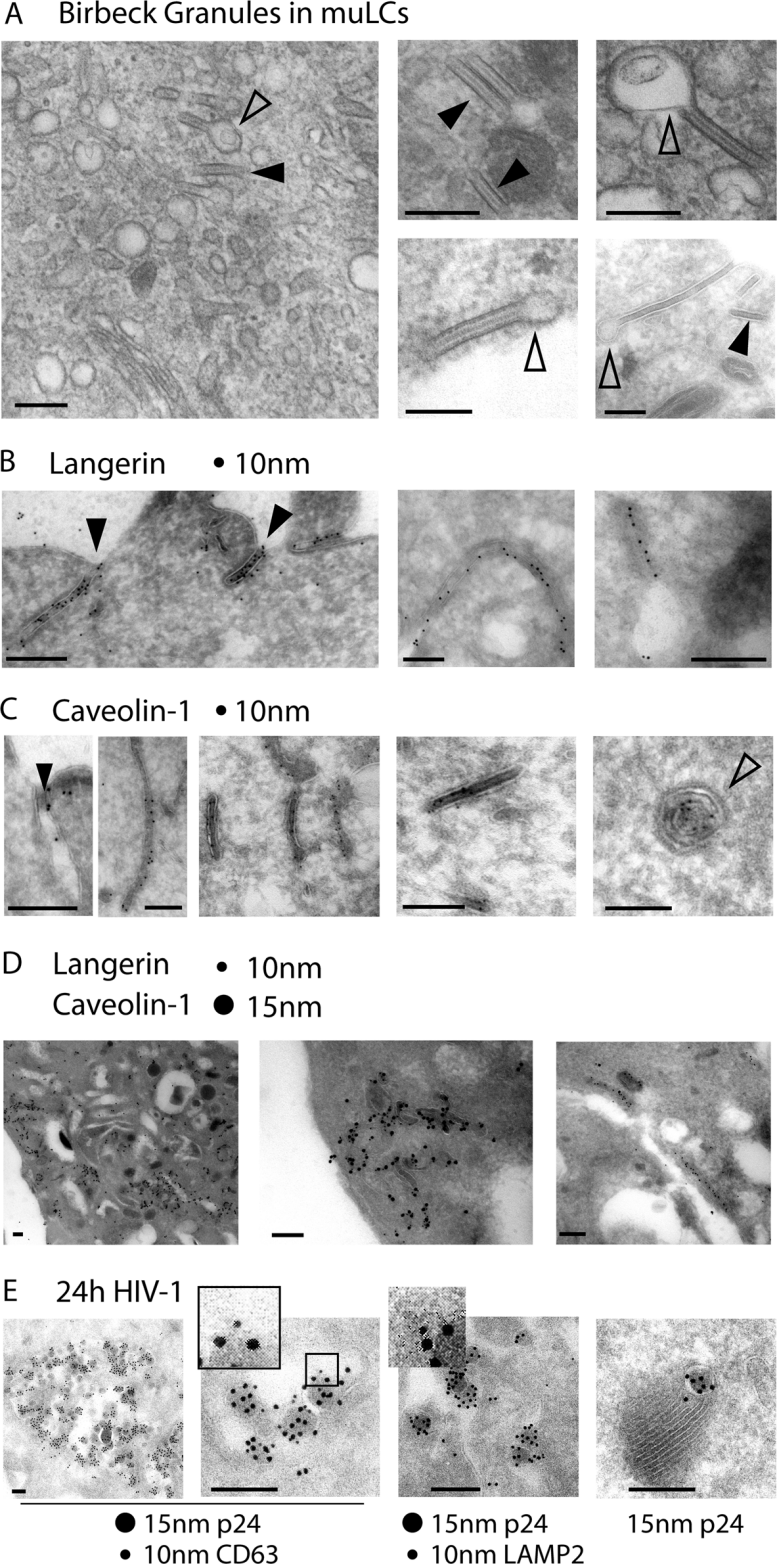
**Langerin and caveolin-1 are present in Birbeck Granules.** MUTZ-LCs were analyzed by immuno-electron microscopy for the presence of BGs that appear tubular (filled arrows) or tennis racquet shaped **(A)**. Sections were stained for langerin with 10 nm gold particles **(B)**, caveolin-1 with 10 nm gold particles **(C)**, langerin and caveolin with 10 nm and 15 nm gold particles, respectively **(D)**. MUTZ-LCs were incubated for 24 hours with HIV-1 and stained for p24-HIV-1 and CD63 or LAMP2 with 10 nm and 15 nm gold particles **(E)**. Bars represent 200 nm and these data are representative of two independent experiments.

We have previously shown that HIV-1 is internalized via langerin into BGs in primary LCs [3]. BGs have been described as rod-shaped structures of variable length with periodically striated lamellae (Figure 3A). Although BGs are suggested to be involved in HIV degradation [3], the exact function of BGs in this process remains unclear. Based on our data, we hypothesized that BGs are part of the caveolin-mediated internalization pathway. We therefore used immuno-transmission electron microscopy to investigate whether caveolin-1 is present in BGs. Because of the paucity in primary LCs we used MUTZ-LCs that have high levels of langerin similar to primary LCs and have been validated as a bona fide LC model [3,29]. MUTZ-LCs expressed high levels of the tennis racquet shaped BGs (Figure 3A, empty arrow heads). Furthermore, depending on the interface and cutting surface, some BGs appeared tubular (Figure 3A, filled arrow heads). BGs originated as invagination in the cell membrane and stained langerin-positive (Figure 3B, filled arrow heads). We noted in particular that caveolin-1 was present in the invaginations of the cell membranes that form BGs (Figure 3C, left panel, filled arrow head). Caveolin-1 was abundant in BGs (Figure 3C, middle panels), which were also positive for langerin (Figure 3B) and appear either tubular (Figure 3B,C) or tennis racquet shaped (Figure 3B right panel). In addition, co-localization of langerin and caveolin-1 was observed not only along the BGs originated as invaginations of the cell membrane but also along the more intracellular BGs (Figure 3D). These data strongly suggest that langerin-mediated internalization into BGs forms part of the caveolar endocytosis pathway. Moreover, caveolin-1 was present in multilaminar lysosomal structures in MUTZ-LCs (Figure 3C, right panel, empty arrow head), which is consistent with previous reports showing that caveolin-1 is targeted via late endosomes to lysosomes for degradation [23]. HIV-1 is taken up via langerin into BGs, which is involved in protection against HIV-1 infection [3,29]. However, the fate of the internalized HIV-1 is unclear. MUTZ-LCs were therefore incubated for 24 hours with HIV-1 and intracellular localization was investigated by staining for lysosomal-associated membrane protein 2 (LAMP2) and the lysosomal tetraspanin CD63. Twenty-four hours post-infection, HIV was observed in LAMP2/CD63 lysosomal vesicles (Figure 3E all panels) suggesting that HIV-1 internalization into BGs intersects the caveolin-1 degradation pathway [23] in LCs.

### Caveolar uptake prevents HIV-1 infection

**Figure 4:**
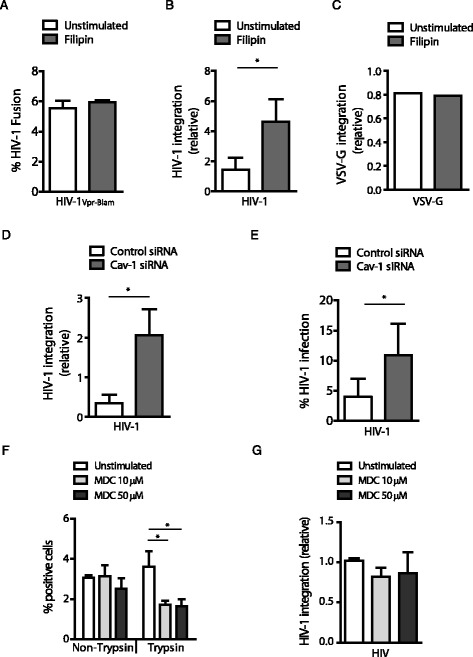
**Inhibition of caveolar uptake increased HIV-1 infection.** LCs were incubated for 2 hours with HIV-1NL4.3/Vpr-BlaM and viral fusion to the host membrane was measured by BlaM assay as described by [31] **(A)**. MUTZ-LCs were incubated with HIV-1 NL4.3-BaL (MOI = 0.2, **B)** or with VSV-G pseudotyped HIV-1 (MOI = 0.2, **C)** in the absence or presence of filipin (1 μg/ml) and integration of HIV-1 DNA was analyzed by *Alu*-PCR. MUTZ-LCs were treated with small interfering RNA (siRNA) of caveolin-1-specific (Cav-1 siRNA) or with non-target control (Control siRNA). Cells were incubated for 18 hours with HIV-1 NL4.3-BaL **(D)** or HIV-1 NL4.3 **(E)**. Integration of HIV-1 DNA was determined by *Alu*-PCR. At day 8 post-infection, HIV-1 infection was measured by intracellular p24 staining. Percentage of CD1a^+^p24^+^ cells are depicted here as % of HIV-1 infection. MUTZ-LCs were incubated with HIV-1 for 4 h in the absence or presence of the clathrin inhibitor monodansylcadaverine (MDC, 10 or 50 μM). Cells were treated with Trypsin-EDTA (0.05%) or left untreated as control. HIV-1 uptake was quantified by permeabilizing cells and measuring subsequent HIV-1-p24 content by flow cytometry **(F)**. MUTZ-LCs were incubated for 18 hours with HIV-1 NL4.3-BaL in the absence or presence of monodansylcadaverine (MDC, 10 or 50 μM) and integration of HIV-1 DNA was analyzed by *Alu*-PCR **(G)**. n = 3 paired students *t*-test; *p < 0.05; SD and mean are depicted **(A, B, F, G)**. One representative experiment out of two is shown **(C)**. n = 4 paired students *t*-test; *p < 0.05; SD and mean are depicted **(D, E)**.

To further elucidate the caveolin-mediated restriction mechanism, we investigated whether routing of HIV-1 into BGs via caveolar internalization specifically inhibits early steps of HIV-1 infection. HIV-1 fusion with the cell membrane and subsequent integration into the host genome are the first steps in HIV-1 infection [30]. We measured HIV-1 fusion to the host membrane 2 hours post-infection using BlaM assay [31] and HIV-1 integration into the genome 6 or 18 hours post-infection using an Alu-PCR integration assay [32]. Blocking caveolar uptake with filipin did not affect HIV-1 fusion (Figure 4A). In contrast, integration of HIV-1 DNA into the host genome increased by blocking caveolar uptake with filipin already at 6 hours post-infection (Additional file 2a), which further expanded to a more than 2-fold increase in integration at 18 hours post-infection (Figure 4B). The increase of HIV-1 integration by filipin treatment was also observed when higher viral loads were used (Additional file 2b). As a control, integration of VSV-G-pseudotyped virus, which does not bind to CD4, CCR5 nor langerin, was not affected by the presence of filipin showing that filipin inhibitor did not interfere with the integration process (Figure 4C). To further confirm the contribution of caveolin-1 to the restriction of HIV-1 integration upon caveolar-mediated uptake, we silenced caveolin-1 by RNA interference (Additional file 2c). Similarly to inhibition of caveolar uptake by filipin, HIV-1 integration was increased after silencing of caveolin-1 (Figure 4D). Furthermore, this increase of HIV-1 integration was accompanied by an increase of HIV-1 infection (Figure 4E). Notably, our data indicate that caveolin-1 mediated internalization restricts HIV-1 infection at a post-entry/pre-integration stage of the HIV-1 cycle in human LCs.

BGs, which partly overlaps with the endosomal recycling pathway [33,34], have been thought to be part of the clathrin-mediated endosomal recycling pathway [12,14]. Therefore, we treated LCs with the clathrin inhibitor monodansylcadaverine (MDC). Inhibition of clathrin-mediated uptake decreased HIV-1 uptake, suggesting that both caveolin- and clathrin-mediated endocytosis are involved in HIV-1 uptake. Strikingly, inhibition of clathrin- in contrast to caveolin-mediated uptake did not affect HIV-1 integration even when higher viral loads were used (Figure 4B,F,G; Additional file 2b). These data suggest that HIV-1 uptake is dependent on both caveolin- and clathrin-mediated endocytosis, but only caveolin-mediated endocytosis restricts HIV-1 infection.

Thus, our data strongly suggest that langerin-positive BGs originate at the cell membrane as langerin^+^caveolin-1^+^ caveolae and subsequently develop into caveolin-1-positive BGs. Both caveolin and clathrin-mediated endocytosis pathways are involved in HIV-1 uptake, however, routing of HIV-1 via caveolar internalization into BGs appears to particularly contribute to the antiviral function of BGs and langerin.

## Conclusions

Altogether, these data strongly indicate an important protective role for caveolar uptake and for the caveolar protein caveolin-1 in limiting HIV-1 infection in LCs. Furthermore, our data show that BGs belong to the caveolar endocytocis pathway and are involved in HIV-1 degradation. Co-infections by sexually transmitted diseases alter the functionality of langerin and increase susceptibility of LCs to HIV-1 infection resulting in HIV-1 transmission to T cells. Novel strategies that strengthen caveolar endocytosis pathway and langerin function have the potential to prevent sexual transmission of HIV-1.

## Methods

### Antibodies and reagents

The following antibodies were used: Rabbit-anti-Caveolin-1 (Cell Signalling); Goat-anti-langerin (R&D); DCGM4-PE (mouse-anti-langerin; Beckman Coulter); KC57-RD1-PE (mouse-anti-p24; Beckman Coulter); mouse-anti-CD1a-FITC (BD Pharmingen); anti-LAMP2 (Abcam); anti-CD63 (BD); sheep-anti-p24 (Aalto); 10E2 (anti-langerin [3]); 10E2 coupled to Alexa-647 (Alexa-647 labeling kit); Streptavidin-Alexa-488; Goat-anti-Mouse IgG_1_ Alexa 546; Goat-anti-Rabbit Alexa 488 (5 μg/ml, all Invitrogen); 15 nm protein A-gold; 10 nm protein G-gold (both Aurion); prot A/G plus agarose beads (Santa Cruz); filipin complex (Sigma Aldrich); Dispase II (Roche Diagnostics); monodansylcadaverine (Sigma Aldrich).

### Donors and cells

Human skin tissue was obtained from healthy donors undergoing corrective breast or abdominal surgery after informed consent in accordance with our institutional guidelines. Split-skin grafts of 0.3 mm were harvested using a dermatome (Zimmer). The slides were incubated with Dispase II (1 U/ml) for 1 hour at 37°C and subsequently the epidermis was mechanically separated from the dermis. Migratory LCs were generated by floating the epidermis onto Iscoves Modified Dulbeccos’s Medium (IMDM) supplemented with 10% FCS, gentamycine (20 μg/ml, Centrafarm), pen/strep (10 U/ml and 10 μg/ml, respectively; Invitrogen) for 2 days. The emigrated cells were layered on a Lymphoprep (1.077 g/ml, Axis-shield) gradient and were routinely 95% pure and expressed high levels of langerin and CD1a. THP-langerin and the CD34^+^ human AML cell line MUTZ3 (MUTZ-LCs) were generated and cultured as described before [3].

### Viruses and infection

HIV-1 NL4.3-BaL, HIV-1 NL4.3, HIV-1 NL4.3/Vpr-BlaM and single round VSV-G pseudotyped virus were generated as previously described [6]. MUTZ-LCs were infected at a multiplicity of infection of 0.2 or 0.6 (Additional file 2). MUTZ-LCs infection was assessed by flow cytometry at day 8 post-infection by intracellular p24 staining. Double staining with CD1a (LCs marker) and p24 was used to discriminate the percentage of CD1a^+^p24^+^ infected LCs.

### Electron microscopy

MUTZ-LCs (3x10^6^) were fixed in 4% paraformaldehyde in 0.1 M phosphate buffer for 1 h at room temperature. Cells were pelleted in 12% gelatine, cryoprotected in 2.3 M sucrose and snap-frozen in liquid nitrogen. Ultrathin cryosections were immunolabeled with Rabbit-anti-Caveolin-1 or Goat-anti-langerin antibody (10 nm protein G gold label; 15 nm potein A gold label). After incubation, the sections were stained with uranylacetate and embedded in 1% methylcellulose. Sections were examined with a transmission electron microscope.

### HIV-1 integration Alu-PCR assay

Total cell DNA was isolated at 6 or 18 hours after infection (multiplicity of infection 0.2) with a QIAamp blood isolation kit (Qiagen) and a two-step Alu-LTR polymerase chain reaction (PCR) was used to quantify the integrated HIV-1 DNA in infected cells as previously described [32] .In the first round of PCR, the DNA sequence between HIV-1 LTR and the nearest Alu repeat was amplified with an HIV-1-specific primer (LTR R region) in combination with a primer that anneals to the abundant genomic Alu repeats. The HIV-1-specific primer was extended with a marker region at the 5’ end, which was used for specificity in the second-round nested real-time quantitative PCR (RT-qPCR). The second round specifically amplified the PCR products from the first-round PCR using primers annealing to the aforementioned marker region in combination with another HIV-1-specific primer (LTR U5 region). Primer sequences were as follows: first round PCR, HIV-1 LTR R forward, 5’-*ATGCCACGTAAGCGAAACTG*CTGGCTAACTAGGGAACCCACTG-3’ (marker sequence in italic); Alu reverse, 5’-TCCCAGCTACTGGGGAGGCTGAGG-3’; second-round RT-qPCR marker forward, 5’-ATGCCACGTAAGCGAAACTG-3’; HIV-1 LTR U5 reverse, 5’-CACACTGACTAAAAGGGTCTGAGG-3’. Two different dilutions of the PCR products from the first-round of PCR were assayed to ensure that PCR inhibitors were absent. For monitoring the signal contributed by unintegrated HIV-1 DNA, the first-round PCR was also performed using the HIV-1-specific primer (LTR R region) only. HIV-1 integration was normalized to GAPDH DNA levels and the results for integration are shown relative to the HIV-infected sample.

### RNA interference

MUTZ-LCs were transfected with 50 nM siRNA with the transfection reagent DF1 (Dharmacon) and were used for experiments 72 h after transfection. The siRNA was specific for Caveolin-1 (M-003467-01; SMARTpool; Dharmacon) and nontargeting siRNA (D-001206-13; Dharmacon) served as control. Silencing of caveolin-1 expression was verified by real-time PCR (Supplementary Figure 2b). Primer sequences were as follows: caveolin-1 forward, 5’-TTTACCGCTTGCTGTCTGCC-3’; caveolin-1 reverse, 5’- GGTACAACTGCCCAGATGTGC -3’; β-actin forward, 5’-GCTCCTCCTGAGCGCAAG-3’ β-actin reverse, 5’- CATCTGCTGGAAGGTGGACA-3’.

### HIV-1 uptake

Cells were incubated for 4 hours with HIV-1 NL4.3 (multiplicity of infection 0.2) in the presence or absence of filipin (1 μg/ml) or monodansylcadaverine (MDC, 10 or 50 μM). Cells were treated with Trypsin-EDTA (0.05%; Invitrogen) or left untreated as control. Cell were fixed with 4% paraformaldehyde and permeabilized with PBS/ 0.5% saponin/1% BSA. Cells were incubated with directly labeled antibody against HIV-1 p24 capsid protein (10 μg/ml) in saponin buffer at 4°C for 30 minutes and samples were analyzed on FACScanto (BD Biosciences).

### Langerin staining

Cells were incubated for 4 hours with filipin (1 μg/ml) or monodansylcadaverine (MDC 50 μM) or left untreated as control. To determine langerin surface expression, cells were incubated with directly labeled antibody against langerin DCGM4-PE (mouse-anti-langerin; Beckman Coulter) in dPBS/1% BSA buffer at 4°C for 30 minutes and samples were analyzed on FACScanto (BD Biosciences). To determine langerin total expression, cells were fixed with 4% paraformaldehyde, permeabilized with dPBS/0.5% saponin/1% BSA. Cells were incubated with directly labeled antibody against langerin in dPBS/0.5% saponin/1% BSA at 4°C for 30 minutes and samples were analyzed on FACScanto.

### Confocal microscopy

Cells were incubated for 4 hours with HIV-1 NL4.3-BaL (multiplicity of infection 0.5) in the presence or absence of filipin (1 μg/ml), followed by fixation with 4% paraformaldehyde and permeabilization with PBS/ 0.1% saponin/1% BSA. Cells were incubated with primary antibody (5 μg/ml) at 4°C for 30 minutes and were subsequently washed 3 times. Then cells were incubated with secondary antibody at 4°C for 30 minutes, washed 3 times, and nuclei were counterstained with Hoechst (10 μg/ml; Invitrogen). Cells were plated onto poly-L-lysine coated slides and single plane images were obtained by a confocal scanning laser microscope (Zeiss).

### Immunoprecipitation and immunoblotting

Whole cell extracts were prepared using RIPA lysis buffer (Cell Signalling). Caveolin and Langerin were immunoprecipitated from 40 μg of extract with anti-Caveolin (Cell Signalling) and 10E2 (anti-langerin [3]) on protein A/G PLUS agarose beads (Santa Cruz). Lysates were resolved by SDS-PAGE, and detected by immunoblotting with Goat-anti-langerin or Rabbit-anti-Caveolin-1 antibodies. This was followed by incubation with HRP-conjugated secondary antibody (Thermo Scientific) and ECL detection (Thermo Scientific).

### Statistical analysis

A paired Student’s *t*-test was used to evaluate the differences of at least 3 donors between filipin-treated or MDC-treated and untreated or between caveolin-1 siRNA treated and control siRNA treated. p < 0.05 was considered significant.

## Additional files

Additional file 1:
**Filipin treatment increased langerin surface expression.** Primary LCs were incubated for 4 h with the caveolar inhibitor filipin (1 μg/ml) or the clathrin inhibitor monodansylcadaverine (MDC, 50 μM). Cells were stained for langerin without or with cell permeabilization and surface langerin expression or total langerin expression was determined, respectively, by flow cytometry (FI, fluorescent intensity). One representative experiment out of three is shown **(A)**. Langerin expression presented relative to langerin surface expression in unstimulated cells, set as 1. n = 3 paired students *t*-test; *p < 0.05; SD and mean are depicted **(B)**. Additional file 2
**Inhibition of caveolar uptake increased HIV-1 integration early post-infection and when using high HIV-1 titers.** MUTZ-LCs were incubated for 6 hours with HIV-1 NL4.3-BaL (MOI = 0.2) and integration of HIV-1 DNA was analyzed by *Alu*-PCR **(A)**. MUTZ-LCs were incubated for 18 hours with higher MOI of HIV-1 NL4.3-BaL (MOI = 0.6) and blocking caveolar uptake induced increase of HIV-1-integration. **(B)** Silencing of caveolin-1 was verified by quantitative real-time PCR at 72 h after transfection **(C)**. Caveolin-1 mRNA levels was normalized to β-actin mRNA levels and the results are shown relative to the control siRNA-treated sample. n = 4 paired students *t*-test; *p < 0.05; SD and mean are depicted. One representative experiment out of two is shown **(A** **B)**.

## Abbreviations

LCs: Langerhans cells
BGs: Birbeck granules
CLR: C-type lectin receptor
MUTZ-LCs: MUTZ3 (CD34^+^ human AML cell line) - derived LCs
MDC: Monodansylcadaverine

## References

[1] van der Vlist M, Geijtenbeek TBH (2010). Langerin functions as an antiviral receptor on Langerhans cells. Immunol Cell Biol.

[2] Cunningham AL, Abendroth A, Jones C, Nasr N, Turville S (2010). Viruses and Langerhans cells. Immunol Cell Biol.

[3] de Witte L, Nabatov A, Pion M, Fluitsma D, De Jong MAWP, de Gruijl T, Piguet V, van Kooyk Y, Geijtenbeek TBH (2007). Langerin is a natural barrier to HIV-1 transmission by Langerhans cells. Nat Med.

[4] Patterson BK, Landay A, Siegel JN, Flener Z, Pessis D, Chaviano A, Bailey RC (2002). Susceptibility to human immunodeficiency virus-1 infection of human foreskin and cervical tissue grown in explant culture. Am J Pathol.

[5] Hladik F, Sakchalathorn P, Ballweber L, Lentz G, Fialkow M, Eschenbach D, McElrath MJ (2007). Initial events in establishing vaginal entry and infection by human immunodeficiency virus type-1. Immunity.

[6] De Jong MAWP, de Witte L, Oudhoff MJ, Gringhuis SI, Gallay P, Geijtenbeek TBH (2008). TNF-alpha and TLR agonists increase susceptibility to HIV-1 transmission by human Langerhans cells ex vivo. J Clin Investig.

[7] Ganor Y, Zhou Z, Tudor D, Schmitt A, Vacher-Lavenu MC, Gibault L, Thiounn N, Tomasini J, Wolf JP, Bomsel M (2010). Within 1 h, HIV-1 uses viral synapses to enter efficiently the inner, but not outer, foreskin mucosa and engages Langerhans-T cell conjugates. Mucosal Immunol.

[8] Sarrami-Forooshani R, Mesman AW, van Teijlingen NH, Sprokholt JK, van der Vlist M, Ribeiro CMS, Geijtenbeek TBH (2014). Human immature Langerhans cells restrict CXCR4-using HIV-1 transmission. Retrovirology.

[9] Valladeau J, Dezutter-Dambuyant C, Saeland S (2003). Langerin/CD207 sheds light on formation of birbeck granules and their possible function in langerhans cells. Immunol Res.

[10] Valladeau J, Ravel O, Dezutter-Dambuyant C, Moore K, Kleijmeer M, Liu Y, Duvert-Frances V, Vincent C, Schmitt D, Davoust J, Caux C, Lebecque S, Saeland S: **Langerin, a novel C-type lectin specific to Langerhans cells, is an endocytic receptor that induces the formation of Birbeck granules.** *Immunity* 2000, **12:**71–81. 10.1016/s1074-7613(00)80160-0 10661407

[11] Thepaut M, Valladeau J, Nurisso A, Kahn R, Arnou B, Vives C, Saeland S, Ebel C, Monnier C, Dezutter-Dambuyant C, Imberty A, Fieschi F: **Structural studies of Langerin and Birbeck granule: a macromolecular organization model.** *Biochemistry* 2009, **48:**2684–2698. 10.1021/bi802151w 19175323

[12] McDermott R, Bausinger H, Fricker D, Spehner D, Proamer F, Lipsker D, Cazenave JP, Goud B, De La Salle H, Salamero J, Hanau D: **Reproduction of Langerin/CD207 traffic and Birbeck granule formation in a human cell line model.** *J Investig Dermatol* 2004, **123:**72–77. 10.1111/j.0022-202X.2004.22728.x 15191545

[13] van der Vlist M, de Witte L, de Vries RD, Litjens M, De Jong MAWP, Fluitsma D, de Swart RL, Geijtenbeek TBH (2011). Human Langerhans cells capture measles virus through Langerin and present viral antigens to CD4(+) T cells but are incapable of cross-presentation. Eur J Immunol.

[14] McDermott R, Ziylan U, Spehner D, Bausinger H, Lipsker D, Mommaas M, Cazenave JP, Raposo G, Goud B, De La Salle H, Salamero J, Hanau D: **Birbeck granules are subdomains of endosomal recycling compartment in human epidermal Langerhans cells, which form where Langerin accumulates.** *Mol Biol Cell* 2002, **13:**317–335. 10.1091/mbc.01-06-0300 PMC65091 11809842

[15] Gallusser A, Kirchhausen T (1993). The beta 1 and beta 2 subunits of the AP complexes are the clathrin coat assembly components. EMBO J.

[16] Owen DJ, Evans PR (1998). A structural explanation for the recognition of tyrosine-based endocytotic signals. Science.

[17] Lapierre LA, Ducharme NA, Drake KR, Goldenring JR, Kenworthy AK (2012). Coordinated regulation of caveolin-1 and Rab11a in apical recycling compartments of polarized epithelial cells. Exp Cell Res.

[18] Polak ME, Thirdborough SM, Ung CY, Elliott T, Healy E, Freeman TC, Ardern-Jones MR (2014). Distinct molecular signature of human skin Langerhans cells denotes critical differences in cutaneous dendritic cell immune regulation. J Investig Dermatol.

[19] Glenney JR, Soppet D (1992). Sequence and expression of caveolin, a protein-component of caveolae plasma-membrane domains phosphorylated on tyrosine in rous-sarcoma virus-transformed fibroblasts. Proc Natl Acad Sci U S A.

[20] Kurzchalia TV, Dupree P, Parton RG, Kellner R, Virta H, Lehnert M, Simons K (1992). Vip21, A 21-Kd membrane-protein is an integral component of Trans-Golgi-network-derived transport vesicles. J Cell Biol.

[21] Fra AM, Williamson E, Simons K, Parton RG (1995). De novo formation of caveolae in lymphocytes by expression of VIP21-caveolin. Proc Natl Acad Sci U S A.

[22] Lipardi C, Mora R, Colomer V, Paladino S, Nitsch L, Rodriguez-Boulan E, Zurzolo C (1998). Caveolin transfection results in caveolae formation but not apical sorting of glycosylphosphatidylinositol (GPI)-anchored proteins in epithelial cells. J Cell Biol.

[23] Hayer A, Stoeber M, Ritz D, Engel S, Meyer HH, Helenius A (2010). Caveolin-1 is ubiquitinated and targeted to intralumenal vesicles in endolysosomes for degradation. J Cell Biol.

[24] Yan M, Peng J, Jabbar IA, Liu X, Filgueira L, Frazer IH, Thomas R (2004). Despite differences between dendritic cells and Langerhans cells in the mechanism of papillomavirus-like particle antigen uptake, both cells cross-prime T cells. Virology.

[25] Smith JL, Campos SK, Ozbun MA (2007). Human papillomavirus type 31 uses a caveolin 1- and dynamin 2-mediated entry pathway for infection of human keratinocytes. J Virol.

[26] Rohde M, Muller E, Chhatwal GS, Talay SR (2003). Host cell caveolae act as an entry-port for group A streptococci. Cell Microbiol.

[27] Orlandi PA, Fishman PH (1998). Filipin-dependent inhibition of cholera toxin: evidence for toxin internalization and activation through caveolae-like domains. J Cell Biol.

[28] Schnitzer JE, Oh P, Pinney E, Allard J (1994). Filipin-sensitive caveolae-mediated transport in endothelium - reduced transcytosis, scavenger endocytosis, and capillary-permeability of select macromolecules. J Cell Biol.

[29] de Jong MA, de Witte L, Santegoets SJ, Fluitsma D, Taylor ME, de Gruijl TD, Geijtenbeek TB (2010). Mutz-3-derived Langerhans cells are a model to study HIV-1 transmission and potential inhibitors. J Leukoc Biol.

[30] Klasse PJ (2012). The molecular basis of HIV entry. Cell Microbiol.

[31] Cavrois M, de Noronha C, Greene WC (2002). A sensitive and specific enzyme-based assay detecting HIV-1 virion fusion in primary T lymphocytes. Nat Biotechnol.

[32] Brussel A, Sonigo P (2004). Evidence for gene expression by unintegrated human immunodeficiency virus type 1 DNA species. J Virol.

[33] Uzan-Gafsou S, Bausinger H, Proamer F, Monier S, Lipsker D, Cazenave JP, Goud B, De La Salle H, Hanau D, Salamero J (2007). Rab11A controls the biogenesis of Birbeck granules by regulating Langerin recycling and stability. Mol Biol Cell.

[34] Gidon A, Bardin S, Cinquin B, Boulanger J, Waharte F, Heliot L, De La Salle H, Hanau D, Kervrann C, Goud B, Salamero J: **A Rab11A/myosin Vb/Rab11-FIP2 complex frames two late recycling steps of langerin from the ERC to the plasma membrane.** *Traffic* 2012, **13:**815–833. 10.1111/j.1600-0854.2012.01354.x 22420646

