# Supplementary figures and images for: Caveolin-1 mediated uptake via langerin restricts HIV-1 infection in human Langerhans cells
Source: Retrovirology. 2014 Dec 31;11:123. doi: 10.1186/s12977-014-0123-7 (PMC4301922; doi:10.1186/s12977-014-0123-7)

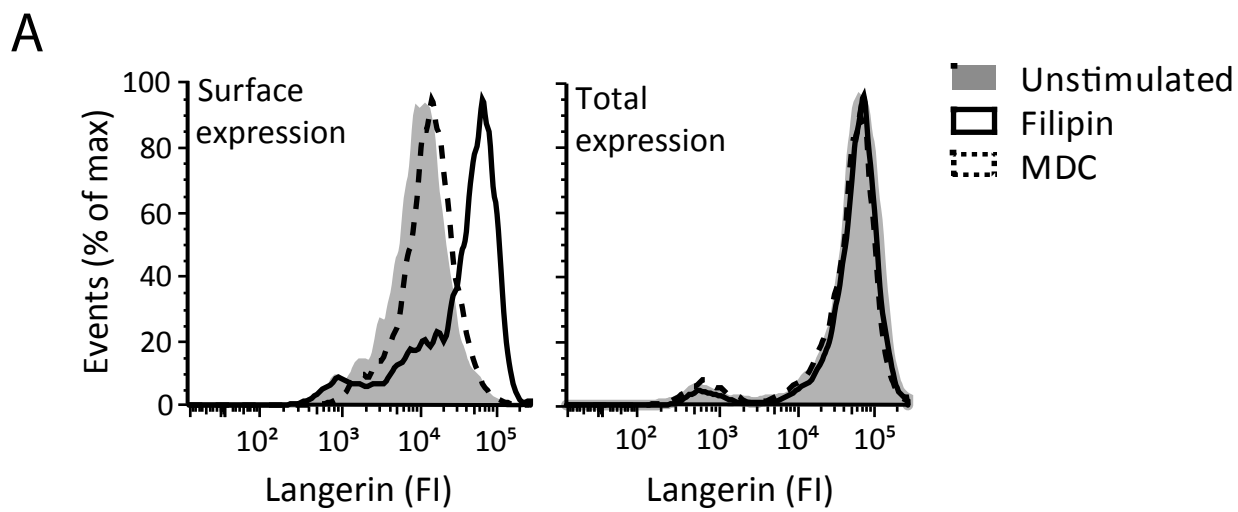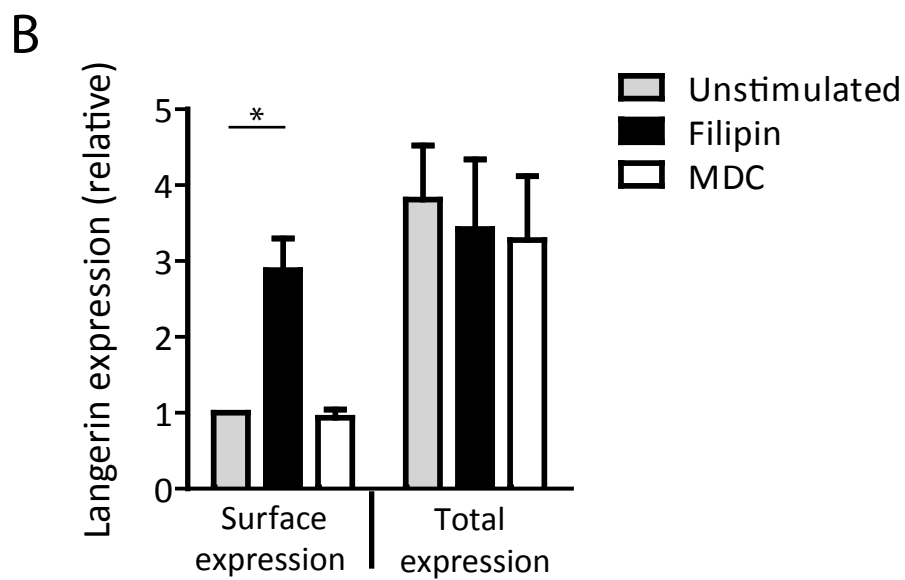

Supplement: Additional file 1: — Filipin treatment increased langerin surface expression. Primary LCs were incubated for 4 h with the caveolar inhibitor filipin (1 μg/ml) or the clathrin inhibitor monodansylcadaverine (MDC, 50 μM). Cells were stained for langerin without or with cell permeabilization and surface langerin expression or total langerin expression was determined, respectively, by flow cytometry (FI, fluorescent intensity). One representative experiment out of three is shown (A). Langerin expression presented relative to langerin surface expression in unstimulated cells, set as 1. n = 3 paired students t-test; *p < 0.05; SD and mean are depicted (B). [file 12977_2014_123_MOESM1_ESM.pdf]

A

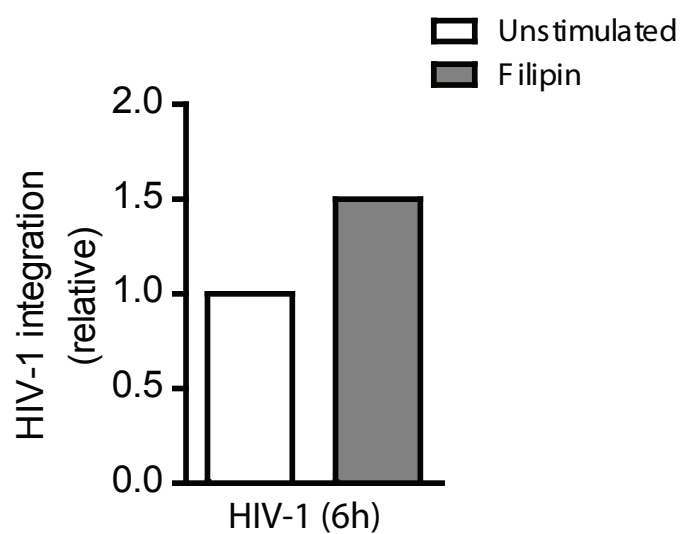

B

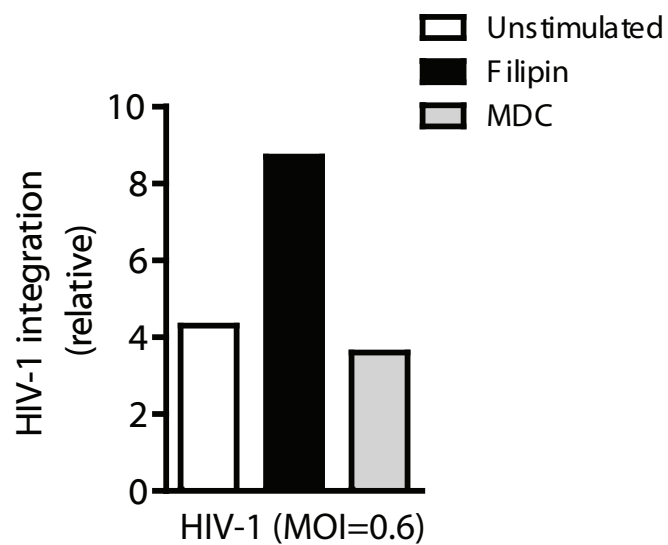

C

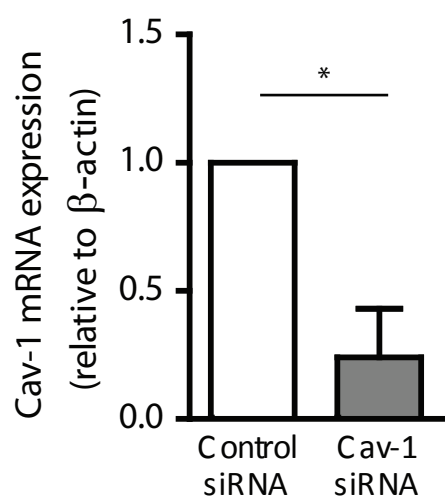

Supplement: Additional file 2 — Inhibition of caveolar uptake increased HIV-1 integration early post-infection and when using high HIV-1 titers. MUTZ-LCs were incubated for 6 hours with HIV-1 NL4.3-BaL (MOI = 0.2) and integration of HIV-1 DNA was analyzed by Alu-PCR (A). MUTZ-LCs were incubated for 18 hours with higher MOI of HIV-1 NL4.3-BaL (MOI = 0.6) and blocking caveolar uptake induced increase of HIV-1-integration. (B) Silencing of caveolin-1 was verified by quantitative real-time PCR at 72 h after transfection (C). Caveolin-1 mRNA levels was normalized to β-actin mRNA levels and the results are shown relative to the control siRNA-treated sample. n = 4 paired students t-test; *p < 0.05; SD and mean are depicted. One representative experiment out of two is shown (A B). [file 12977_2014_123_MOESM2_ESM.pdf]
